# Cirrhosis, Operative Trauma, Transfusion, and Mortality: A Multicenter Retrospective Observational Study

**DOI:** 10.7759/cureus.3087

**Published:** 2018-08-02

**Authors:** Claire Isbell, Stephen M Cohn, Kenji Inaba, Terence O’Keeffe, Marc De Moya, Seleshi Demissie, Mira Ghneim, Matthew L Davis

**Affiliations:** 1 Surgery, Temple, Texas, USA; 2 Surgery, Staten Island University Hospital, Queens Village, USA; 3 Surgery, LA County, Los Angeles, USA; 4 Surgery, The University of Arizona Medical Center, Tucson, USA; 5 Surgery, Medical College of Wisconsin, Wisconsin, USA; 6 Research, Intermountain Healthcare, Staten Island, USA

**Keywords:** acidemia, blood transfusion, cirrhosis, coagulopathy, exploratory laparotomy.

## Abstract

Background: In trauma patients with cirrhosis who require laparotomy, little data exists to establish clinical predictors of the outcome. We sought to determine the prognosticators of mortality in this population.

Methods: We performed a 10-year review at four, busy Level I trauma centers of patients with cirrhosis identified during trauma laparotomy. We compared vital signs, laboratory values, and transfusion requirements for those who survived versus those who died. A linear regression was then conducted to determine the variables associated with death in this population.

Results: A total of 66 patients were included and 47% (31/66) died. The model for end-stage liver disease (MELD) score was low (7.8 in Lived, 10.2 in Died). Packed red blood cell (PRBC) transfusion at six hours was greater in those who died; those receiving > 6 units of PRBCs at 6 hours had an increased likelihood of death (odds ratio OR 5.8 (95% CI 1.9, 17.4)). All patients receiving ≥ 17 units of PRBCs died. We found an association between lower preoperative platelets (PLTs), higher preoperative international normalized ratio (INR) and partial thromboplastin time (PTT), lower preoperative pH (presence of profound acidemia), increased intraoperative crystalloid use, and increased intraoperative blood product administration to be associated with death (p < 0.05).

Conclusions: Cirrhotic trauma patients requiring laparotomy should be considered to have a high chance of mortality if they receive six or more PRBCs, are acidotic (pH ≤ 7.25) at the time of hospital arrival, or have coagulopathy at the time of admission (INR > 1.2, PTT > 40).

## Introduction

The death rate for patients with cirrhosis following surgery is quite high (9% for elective surgery; 47% for emergent cases) [1-2]. The mortality rate in trauma patients with cirrhosis undergoing laparotomy appears to be even higher, ranging from 40%-56% depending upon the severity of injury [3-6]. In addition, this group has an exceedingly high complication rate (71%) and a prolonged length of hospital stay [3]. Reducing mortality has been challenging due to risk factors, such as severity of liver disease, which are not modifiable.

It appears that a small subset of trauma patients receive the majority of blood transfusions. Three percent of the trauma population receive nearly 75% of the blood transfused (144 of 5632 trauma patients at one Level I facility were infused with 72% of the blood administered) [7]. While cirrhotic trauma victims represent only approximately 1% of all trauma patients, they represent a challenging population to resuscitate, consuming a large amount of blood bank resources: mean of 14-19 units of packed red blood cells (PRBCs) and fresh frozen plasma [8]. Trauma patients with cirrhosis may exhibit a coagulopathy at baseline as evidenced by an increased admission international normalized ratio (INR).

The association of anemia, coagulopathy, and model for end-stage liver disease (MELD) score with mortality has not been investigated in cirrhosis and a concurrent abdominal exploration for trauma. We examined the outcomes of patients requiring laparotomy where cirrhosis was diagnosed during operation, in an effort to delineate risk factors for mortality.

## Materials and methods

We performed a retrospective chart review at four busy Level I trauma centers. Each center received approval from their individual institutional review boards to conduct the study.

We reviewed trauma registries during a 10-year time period from January 1, 2001, to December 31, 2010, for trauma patients undergoing an exploratory laparotomy. We identified patients undergoing emergent laparotomy for trauma. Operative reports were examined for the physical finding of cirrhosis and these patients were included in our analysis.

Demographic and clinical data were collected, including mechanism of injury, admission vital signs, laboratory studies (pre- and postoperative), operative details, mortality, and blood product usage at 6, 24, and 48 hours after injury. The model for end-stage liver disease (MELD) score was calculated retrospectively from preoperative laboratory values. Laboratory data were missing for some subjects due to the retrospective nature of this study. Descriptive statistics were used for traumatic mechanism and demographic data. Other comparisons were conducted comparing the groups who “Lived” vs. those who “Died.” Continuous variables were assessed for statistical differences using the Student’s t-test or the Wilcoxon rank-sum for parametric and nonparametric data, respectively. Linear regression was then used to determine which variables—specifically those related to coagulopathy and transfusion requirement—were associated with death in this population. Odds ratios were reported for the variables associated with death.

## Results

A total of 66 subjects were collected from four study sites during the 10-year time period. The subjects were mostly male 62/65 (95%) and white 53/65 (82%) with a median age of 49 years (interquartile range (IQR) 44-56). The mechanism of action of most (70%) of the injured individuals was blunt with motor vehicle collisions as the most common type of blunt trauma (24%). Stab wounds were the most common cause of penetrating injuries (24%) while gunshot wounds made up only 6% of the study patients. Other blunt mechanisms included auto-pedestrian (20%), falls (11%), assaults (9%), motorcycle collisions (3%), and auto-bicycle (1.5%).

Thirty-one of the total 66 subjects died during their hospitalization for a mortality rate of 47%. Fourteen subjects died within the first 24 hours of presentation (45% of all deaths). Four patients did not survive past the operating room. For those who died within 24 hours, the most common cause of death was a hemorrhagic shock. Fifteen percent (10/66) of the subjects had specifically reported liver injuries. Of these 10 subjects, 60% (6/10) died. The median hospital length of stay was five days (IQR 1-26) for those who died and 14 days (IQR 6-25) for those who survived. Late deaths were attributed to multisystem organ failure and cardiac arrest.

Results for admission vital signs, Glasgow coma scale (GCS), and injury severity score (ISS) are seen in Table 1.

**Table 1:** Admission vital signs and injury severity in trauma patients with cirrhosis undergoing laparotomy Systolic blood pressure (SBP); respiratory rate (RR); Glasgow coma scale (GCS); injury severity score (ISS)

Parameter*	Lived Median (IQR)	n	Died Median (IQR)	n	p-value
Heart rate (bpm)	89 (74-112)	34	103 (87-117)	29	0.1
SBP (mm Hg)	130 (110-144)	27	110 (86-133)	25	0.01
RR (breaths/min)	20 (18, 22)	33	20 (18, 24)	23	0.12
GCS	15 (14-15)	33	12 (3-15)	30	0.01
ISS	14 (9-26)	32	21 (17-34)	26	0.03

Mentation was significantly worse in those who died compared to survivors (GCS 12 vs. 15, p<0.05) and the injury burden was significantly greater in those who died (ISS 27 (IQR 17-41) vs. 17 (IQR 9-29), p < 0.05]. Table 2 reveals admission laboratory values. MELD was noted to be low overall in all subjects (median 7.8 in those who lived and 10.2 in those who died).

**Table 2:** Lab values at the time of admission Hemoglobin (Hgb); white blood cells (WBC); international normalized ratio (INR); partial thromboplastin time (PTT); model for end-stage liver disease (MELD)

Lab value*	Lived Median (IQR)	n	Died Median (IQR)	n	p-value
Hgb (g/dL)	11 (9.8-13.1)	32	10.0 (8.5-11)	29	0.03
WBC(1x 10^3^)	11.2 (7-15.6)	32	9.1 (6.2-10.5)	29	0.09
Platelets ( 1x 10^3^)	157 (114-210)	35	93 (72-179)	30	0.04
Sodium (mEq/L)	141 (138-145)	32	141 (134-143)	30	0.76
Creatinine (mg/dL)	1.0 (0.9-1.2)	32	1.0 (0.8-1.3)	29	0.52
Albumin (g/dL)	2.9 (2.5-3.3)	26	2.35 (1.85-3)	20	0.08
Bilirubin, total	0.6 (0.3-1.15)	28	0.6 (0.5-1.1)	22	0.62
Lactate (mmol/L)	2.4 (1.9-8.4)	7	6.4 (2.7-9.5)	10	0.33
HCO_3_ (mmol/L)	18.4 (14.1-22)	18	20 (18-22)	21	0.21
Base excess (mEq/L)	-6.7 (-4- -13.6)	10	-14 (-6- -17)	9	0.21
pH	7.34 (7.3-7.38)	15	7.14 (7.04-7.3)	15	0.002
INR	1.2 (1.15-1.3)	30	1.5 (1.3-1.85)	27	0.0002
PTT	31 (IQR 28-34	25	42 (IQR 35-73)	18	<0.0001
MELD	7.8 (5.1-10.9)	26	10.2 (6.6-12.1)	20	0.29

Of note, patients who died had a statistically significantly lower admission serum hemoglobin value and platelet count (93 vs. 157) and a higher INR (median 1.5 vs. 1.2).

Table 3 shows other variables after admission for all subjects.

**Table 3:** All variables (except admission) for all subjects comparing "lived" vs. "died" Estimated blood loss (EBL); packed red blood cells (PRBCs); intensive care unit (ICU); mean arterial pressure (MAP); international normalized ratio (INR); partial thromboplastin time (PTT)

Variable	Lived	n	Died	n	p-value
Intraoperative					
Operative time (minutes)	108 (IQR 75-150)	31	103 (IQR 70-139)	27	0.73
EBL (cc)	1500 (IQR 500-3000)	23	2500 (IQR 400-5000)	23	0.37
Crystalloid (cc)	3200 (2000-5000)	22	5000 (2000-10,000)	21	0.29
PRBCs (units)	1 (0-4)	35	7 (2-18)	30	0.002
Postoperative					
ICU admission	80%	28	83%	25	0.02
MAP (mm Hg), ICU	86 (76-107)	26	77 (54-88)	21	0.01
Platelet count (1x10^3^)	111 (IQR 78-139)	27	87 (IQR 56-126)	21	0.13
INR	1.32 (IQR 1.20-1.50)	26	1.59 (IQR 1.4-2.0)	22	0.006
INR, 24 hrs postoperative	1.23 (1.15-1.34)	22	1.38 (1.32-1.70)	12	0.005
INR, 48 hrs postoperative	1.23 (1.18-1.39)	20	1.54 (1.24-1.80)	14	0.04
Sodium	143 (IQR 140-146)	27	143 (IQR 137-148)	23	0.95
Creatinine	0.9 (IQR 0.70-1.0)	27	0.9 (IQR 0.8 – 1.1)	22	0.27
PTT	33 (IQR 28-37)	25	46 (IQR 33-59)	22	0.008
HCO_3_	21 (IQR 17-24)	25	20 (IQR 17-23)	22	0.32
Albumin, postoperative	2.7 (IQR 2.5-3.0)	15	2.0 (1.5-2.6)	17	0.01
Albumin, 24 hrs postoperative	2.9 (2.5-3.2)	19	2.4 (2.2-2.6)	7	0.03
Albumin, 48 hrs postoperative	2.7 (2.4-3.0)	19	2.2 (2.0-2.8)	9	0.1
Bilirubin, total, postoperative	0.9 (0.7-1.6)	15	1.15 (0.5-2.5)	18	0.65
Bilirubin, total, 24 hrs postoperative	1.5 (1.10-2.20)	19	2.0 (1.4-4.5)	7	0.18
Bilirubin, total, 48 hrs postoperative	1.7 (1.0-3.5)	20	2.6 (1.5-7.6)	9	0.16
Lactate	3.6 (2.8-6)	21	8.3 (4.2-12.7)	18	0.006
pH, postoperative	7.35 (7.25-7.44)	26	7.24 (7.13-7.35)	24	0.006
pH, 24 hrs postoperative	7.42 (7.4-7.47)	22	7.4 (7.38-7.41)	14	0.11
pH, 48 hrs postoperative	7.39 (7.37-7.45)	19	7.41 (7.35-7.42)	14	0.53
Base excess (mEq/L)	-3.4 (0.5- -7)	22	-9 (-6- -10.5)	17	0.003
Base excess (mEq/L), 24 hrs postoperative	2.2 (0.6-3.10)	17	-4 (-7.8- 4.0)	11	0.06
Base excess (mEq/L), 48 hrs postoperative	3.25 (1.7-6.25)	16	0.0 (-4.30-3.7)	11	0.04

Those who died had more blood transfused during surgery, more frequent intensive care unit (ICU) admission, lower mean arterial pressure (MAP) after surgery, a higher INR up to 48 hours after surgery, a higher PTT after surgery, a lower albumin up to 24 hours after surgery, a higher lactate level, a lower pH, and a lower base excess up to 48 hours after surgery (p < 0.05).

Blood product usage at six hours is seen in Table 4.

**Table 4:** Blood product usage within six hours of admission Packed red blood cells (PRBCs); fresh frozen plasma (FFP)

Blood product* (units)	Lived (n=35) Median (IQR)	Died (n=31) Median (IQR)	p-value
Six hours after presentation:			
PRBC	2 (0-7)	10 (3-22)	<0.001
FFP	0 (0-6)	8 (2-16)	<0.001
Platelets	0 (0-1)	0 (0-3)	0.22

**Figure 1:**
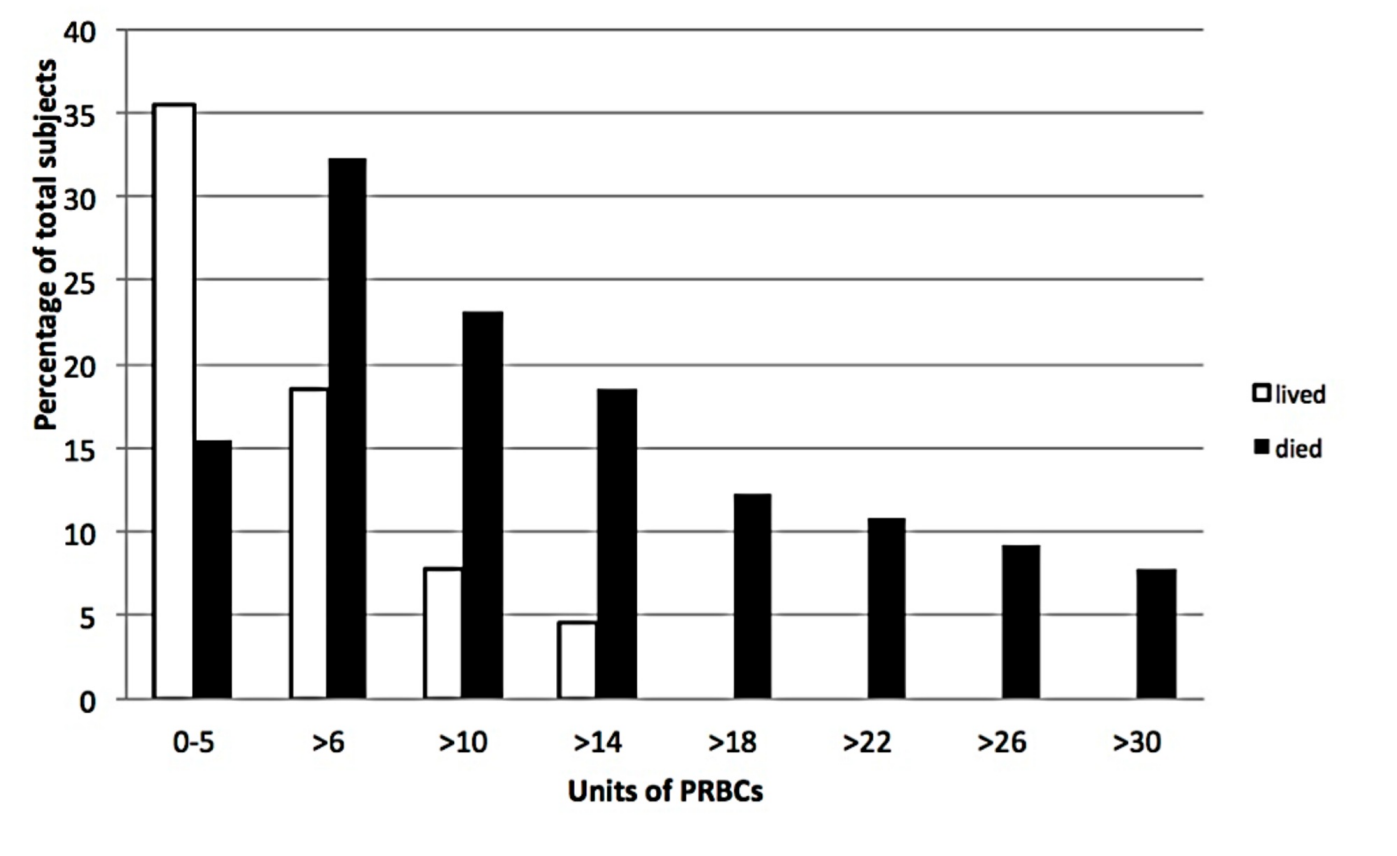
PRBC administration at six hours Packed red blood cells (PRBCs)

Mean packed red blood cells received was significantly greater in patients who died versus those who lived at six hours (10 units (IQR 3-22) vs. 2 units (IQR 0-7), p < 0.01). Those who lived received fewer transfusions than those who died (median 0.5 vs. 7 units, p < 0.01). The spread of all PRBCs transfused during the entire hospitalization between those who lived vs. those who died is seen in Figure 1.

No subject receiving greater than 17 units of PRBCs survived.

The odds ratios for variables associated with death are seen in Table 5.

**Table 5:** Odds ratios of death for all variables examined Note that all were significant except PRBCs transfused at 24 hours. International normalized ratio (INR); partial thromboplastin time (PTT); packed red blood cells (PRBCs)

Variable	Odds Ratio	95% CI	p-value
pH ≤ 7.25 (admission)	0.13	0.03-0.66	0.03
Platelet count ≤ 100 (admission)	0.09	0.02-0.38	0.0004
INR > 1.2 (admission)	6.3	1.86-21.31	0.003
PTT > 40 (admission)	30	3.3-272.3	0.0002
PTT > 40 (postoperative)	6.8	1.88-24.5	0.003
PRBCs transfused > 6 units (6 hours)	5.8	1.9-17.4	0.002
PRBCs transfused > 10 units (24 hours)	2.85	0.96-8.51	0.07

Acidemia (pH ≤ 7.25), INR > 1.2, admission PTT > 40, postoperative PTT > 40, and PRBCs > six units transfused at six hours after admission were all associated with a higher risk of death. A multivariate analysis with the variables seen in Table 5 only showed that INR > 1.2 remained in the model. Admission and postoperative PTT were not included in the model, as many subjects had these variables missing.

No statistical differences between those patients who died when compared to those who survived were seen in operative time, estimated blood loss, intraoperative fluid administration, or PRBCs > 10 units transfused at 24 hours after admission.

## Discussion

To date, this study is unique in that it is the only one of its kind looking specifically at cirrhotic trauma patients with undergoing laparotomy, without preoperative imaging or a known diagnosis of cirrhosis. We investigated this population because we wanted to re-create the “real-life” challenging situation trauma surgeons experience when they take a patient in extremis to the operating room for laparotomy, only to find that the liver is cirrhotic and tremendous resources are being consumed during a heroic resuscitation. Using data from multiple centers, we show that lower platelet count and worsened coagulopathy are associated with mortality in cirrhotic patients with operative trauma. In addition, transfusion of PRBCs ≥ 6 units at six hours significantly increased the likelihood of mortality.

Previous investigators have used markers such as MELD and Child-Pugh scoring to predict operative outcomes in cirrhotic patients [9-11]. MELD was originally created to predict 30-day mortality after a TIPS procedure in a liver transplant candidate but has become a marker for severity of liver disease and is currently used to prioritize patients on the transplant list [12]. MELD has some limitations when calculated in the acute setting, as some of the components in the injured patient may be different from the patient’s uninjured baseline. Nonetheless, it has increasingly been used as a marker by which physicians can qualify the severity of liver disease in those patients undergoing non-liver surgery. Inaba and colleagues have demonstrated that MELD has a strong association with mortality in cirrhotic trauma patients (area under the receiver operating characteristic curve (AUC ROC) = 0.944) [13]. Lin et al. reported that patients with MELD ≥ 17 are at high risk for postoperative death [14]. Our study population had a relatively low median MELD of 10.2 with a predicted baseline mortality of 1.9%-6%, yet our mortality rate was still quite high (47%), leading us to believe that other factors, such as associated injury, shock state, and clotting ability, may lead to death in this patient population [15].

Another study of 68 trauma patients with cirrhosis treated between 2003-2008 determined Child-Pugh, rather than MELD, to be a better indicator of hepatic complications and survival [16]. The real-time calculation of the Child-Pugh score becomes problematic in a patient who is obtunded on arrival with a concomitant head injury or substance abuse or in whom care has not been previously established, as components of the score (presence of encephalopathy and ascites) are unknown. For this reason, calculating this score in our study was not possible.

Neeff and colleagues found that the presence of emergent surgery and perioperative transfusion and the severity of liver disease were predictors of worse outcome [1]. Likewise, our results show that the quantity of blood received can portend mortality in this high-risk population. According to our data, the presence of blood transfusion > six units shows a significant odds ratio for death as early as six hours after hospital presentation. This may be a function of baseline coagulopathy and anemia, as these factors were also associated with mortality in our patients. Additionally, we found that acidemia at the time of admission is associated with mortality. Acidemia (pH < 7.20) may contribute to worsened trauma-induced coagulopathy and mortality, as previously described [17-18].

Limitations

The limitations of our study lie in the low incidence of cirrhotic patients who undergo a laparotomy after trauma. For this reason, our study spans a period of 10 years, during which blood transfusion practices changed significantly. With the advent of massive transfusion protocols (MTPs), the total quantity of blood transfused to patients has changed and, therefore, the actual threshold of transfusion above which death is imminent might be different from 17 units of PRBCs total or six units at six hours. Due to the retrospective nature of this study, data points (i.e. base deficit (BD)) in some patients were missing. This limited our ability to fully characterize shock in the entire population. No patient with BD < -14 survived, albeit the numbers were small (only 16 subjects had a recorded BD on arrival). We commonly use BD as a marker of shock and, when it normalizes, a marker of the adequacy of resuscitation (in our study we used pH as a surrogate). Given this information, the usage of MTP should be encouraged in all trauma patients, but the endpoints (i.e., limits) used to guide resuscitation in cirrhotics may need to be readdressed.

Finally, early death may be associated with transfusion quantity, coagulopathy, or shock state prior to six hours after admission. Unfortunately, we do not have data prior to that time and after admission. This is a question to be addressed in future research.

## Conclusions

In summary, patients with cirrhosis after severe trauma have a high risk of mortality, even in the setting of low MELD. The presence of coagulopathy, lower hemoglobin, lower platelet count, and lower pH (acidemia) increases the risk of death. Transfusion of six or more units of PRBCs is associated with mortality, specifically early (< 24 hours after admission) in the hospital course. Aggressive correction of these factors and resuscitation remain the mainstay of treatment for these complicated patients.
